# Performance Metrics for Selecting Single Nucleotide Polymorphisms in Late-onset Alzheimer’s Disease

**DOI:** 10.1038/srep36155

**Published:** 2016-11-02

**Authors:** Yen-Ching Chen, Chi-Jung Hsiao, Chien-Cheng Jung, Hui-Han Hu, Jen-Hau Chen, Wen-Chung Lee, Jeng-Min Chiou, Ta-Fu Chen, Yu Sun, Li-Li Wen, Ping-Keung Yip, Yi-Min Chu, Chien-Jen Chen, Hwai-I Yang

**Affiliations:** 1 Institute of Epidemiology and Preventive Medicine, College of Public Health, National Taiwan University, Taipei, Taiwan; 2 Department of Public Health, College of Public Health, National Taiwan University, Taipei, Taiwan; 3 Research Center for Genes, Environment and Human Health, College of Public Health, National Taiwan University, Taipei, Taiwan; 4 Genomics Research Center, Academia Sinica, Taipei, Taiwan; 5 Department of Geriatrics and Gerontology, National Taiwan University, Taipei, Taiwan; 6 Institute of Statistical Science, Academia Sinica, Taipei, Taiwan; 7 Department of Neurology, National Taiwan University Hospital, Taipei, Taiwan; 8 Department of Neurology, En Chu Kong Hospital, Taipei, Taiwan; 9 Department of Laboratory Medicine, En Chu Kong Hospital, Taipei, Taiwan; 10 Center of Neurological Medicine, Cardinal Tien Hospital, Taipei, Taiwan; 11 School of Medicine, Fu-Jen Catholic University, New Taipei City, Taiwan; 12 Department of Laboratory Medicine, Cardinal Tien Hospital, Taipei, Taiwan; 13 Institute of Clinical Medicine, National Yang-Ming University, Taipei, Taiwan

## Abstract

Previous genome-wide association studies using *P-*values to select single nucleotide polymorphisms (SNPs) have suffered from high false-positive and false-negative results. This case-control study recruited 713 late-onset Alzheimer’s disease (LOAD) cases and controls aged ≥65 from three teaching hospitals in northern Taiwan from 2007 to 2010. Performance metrics were used to select SNPs in stage 1, which were then genotyped to another dataset (stage 2). Four SNPs (*CPXM2* rs2362967, *APOC1* rs4420638, *ZNF521* rs7230380, and rs12965520) were identified for LOAD by both traditional *P*-values (without correcting for multiple tests) and performance metrics. After correction for multiple tests, no SNPs were identified by traditional *P*-values. Simultaneous testing of *APOE* e4 and *APOC1* rs4420638 (the SNP with the best performance in the performance metrics) significantly improved the low sensitivity of *APOE* e4 from 0.50 to 0.78. A point-based genetic model including these 2 SNPs and important covariates was constructed. Compared with elders with low-risks score (0–6), elders belonging to moderate-risk (score = 7–11) and high-risk (score = 12–18) groups showed a significantly increased risk of LOAD (adjusted odds ratio = 7.80 and 46.93, respectively; *P*_trend_ < 0.0001). Performance metrics allow for identification of markers with moderate effect and are useful for creating genetic tests with clinical and public health implications.

Dementia is an important health issue in the elderly (aged ≥65 years). Alzheimer’s disease (AD) is the most common subtype of dementia and cannot currently be cured, prevented, or even slowed^1^. In 2010, the prevalence of AD among the elderly in the United States was 11%, and the disease was the fifth leading cause of death in this age group^2^. Taiwan has a very high aging rate^3^, and a large survey conducted between 2011 and 2012 showed that the prevalence of dementia was 4.84% in the elderly^4^. Several risk factors, including genetic factors, old age, female gender, vascular risk factors, life style, social factors, and environmental exposure, have been related to AD^5^. Among various genetic factors, single-nucleotide polymorphisms (SNPs), which are germline polymorphisms that remain stable throughout life, are particularly suitable for predicting disease risk at an early stage.

Apolipoprotein E (*APOE*) e4 has been associated with both familial and sporadic AD since 1993^6^ and is the only genetic marker strongly associated with late-onset AD (LOAD). A meta-analysis showed that one *APOE* e4 allele (e4/e2 or e4/e3) was associated with a 2.7-fold (Caucasian) to 5.6-fold (Japanese) increased risk of AD; this risk increased further in people carrying two *APOE e4* alleles (11.8-fold for Caucasians, 33.1-fold for the Japanese)^7^. Despite the strong association between *APOE* e4 status and the disease, the sensitivity of *APOE* e4 for predicting AD risk is only approximately 0.4^8^, which indicates it has limited clinical implications.

In the past decade, genome-wide association studies (GWASs) have been widely conducted to identify genetic markers for AD^9^,^10^,^11^. GWASs in east-Asian populations are limited; only one Japanese GWAS^12^ has been conducted. Although several studies in China have validated SNPs identified from GWASs in the western countries^13^,^14^,^15^, no GWAS has been conducted in a Chinese population using microarray chips customized for Chinese populations. For LOAD, some genes have been consistently identified across GWASs, and these genes mainly belong to three biological pathways, i.e., the metabolic, trafficking, and signaling pathways^16^,^17^,^18^. These genes include *APOE* e4*, CLU, CR1, PICALM, B1N1, ABCA7*, the *MS4A* gene cluster, *CD2AP, CD33, EPHA1*, and *TREM2*^19^,^20^,^21^,^22^,^23^,^24^. However, studies applying traditional *P-*values to identify genetic markers for various research outcomes have suffered from high false-positive and false-negative results^25^,^26^. Hence, these studies are unable to identify SNPs with moderate effects. In addition, these studies have mainly focused on Caucasians and may not be representative of other ethnic groups. Therefore, a recent trend of genetic analysis for resolving the issue of SNP selection includes approaches using high-dimensional multivariable modeling (e.g., penalized logistic regression and Bayesian analysis), clinical validity [i.e., sensitivity, specificity, positive predictive value (PPV), and negative predictive value (NPV)], etc.^27^,^28^,^29^.

Clinical validity (i.e., sensitivity, specificity) has been proposed to be a more practical measure for selecting SNPs in GWASs^30^ and is important for evaluating and translating genetic tests for public health and clinical implications (Centers for Disease Control and Prevention, USA) (http://www.cdc.gov/genomics/gtesting/ACCE/). However, to the best of our knowledge, no GWAS has yet used clinical validity and other performance metrics [e.g., sensitivity, specificity, Youden index, PPV, NPV, diagnostic odds ratio (DOR), accuracy, net sensitivity, net specificity, and area under the receiver operating characteristic curve (AUC)] to identify SNPs for health outcomes. In this study, a two-stage design was adopted, and performance metrics were used to identify SNPs for predicting LOAD. Although *APOE* e4, which is determined by rs429358 and rs7412, is the best-known and strongest genetic factor for LOAD, it suffers from low sensitivity (0.4) for discriminating LOAD^8^. Simultaneous testing of *APOE* e4 and the identified SNPs was applied here in an attempt to improve sensitivity. In addition, a point-based genetic model including these two SNPs and important covariates was constructed to differentiate elders with low, moderate, and high risk of LOAD.

## Results

### Characteristics of the study population

After restriction and matching between the LOAD cases and controls in stage 1 (training set, *n* = 94), the distributions of age, years of education, and the *APOE* e4 status were significantly different between LOAD and the control groups (Table 1). In stage 2 (validation set, n = 619), age, years of education, body mass index (BMI, kg/m^2^), sex, *APOE* e4 status, alcohol consumption, smoking status, history of stroke, diabetes mellitus (DM), and hypercholesterolemia significantly differed between the LOAD and control groups.

### Identification of SNPs in stage 1 (Training set)

In stage 1, 858 SNPs deviated from Hardy-Weinberg Equilibrium (HWE) tests and were excluded from further analysis. After quality control, the quantile-quantile (QQ) plot for the observed chi-square *P*-values of 500,941 SNPs revealed no deviation from the expected values, i.e., there was no significant association with the AD outcome as demonstrated by the diagonal line (Figure S1). Five SNPs (*CCDC81* rs10501617, *CPXM*2 rs2362967, *APOC1* rs4420638, *ZNF521* rs7230380, and rs12965520) with the best performance (the largest number of performance metrics with the highest value) among the performance metrics were selected for genotyping in stage 2 (Table 2). The performance of these five SNPs in stage 1 was as follows: sensitivity (0.56–0.89), specificity (0.49–0.90), Youden index (0.38–0.46), PPV (0.71–0.89), NPV (0.60–0.78), DOR (6.4–11.6), accuracy (0.71–0.74), net sensitivity (0.78–0.95), net specificity (0.44–0.81), and AUC (0.860–0.888, Table 2).

For comparison with traditional GWASs, a Manhattan plot (Figure S2) was produced to assess the distribution of *P-*values by chromosome. Without correction for multiple tests, the top six SNPs (because of ties) identified by traditional *P*-value were *CPXM*2 rs2362967, *APOC1* rs4420638, *ZNF521* rs7230380 and rs12965520 and *BHLHB2* rs2137946 and rs2137947. No single SNP was significantly associated with LOAD after correction for multiple tests because the *P-*values of these top six SNPs ranged from 1.3 × 10^−4^ to 6.2 × 10^−6^ (Table 2), and these values were larger than the Bonferroni-corrected *P-*values (*α*/number of SNPs = 10^−7^).

### Construction of the genetic prediction model in stage 2 (Validation set)

The top five SNPs identified by performance metrics in stage 1 were genotyped in stage 2 (validation set). These SNPs showed no significant deviation from HWE among the controls (n = 423) after correction for multiple tests, and their minor allele frequencies (MAFs, 11–50%) were similar to those in the Han Chinese in Beijing from the HapMap dataset (12–50%, Table 3). Because of the strong effect of *APOE* e4 on LOAD risk observed in this study [e3/e4 vs. e3/e3: adjusted odds ratio (AOR) = 3.5; e4/e4 vs. e3/e3: AOR = 17.7] and a previous meta-analysis of Japanese subjects [e3/e4 vs. e3/e3: odds ratios (OR) = 3.9; e4/e4 vs. e3/e3: OR = 21.8]^7^, *APOE* e4 status was forced into the genetic model for AD. In stage 2, among the top five SNPs, *APOC1* rs4420638 showed the best overall performance in performance metrics, especially in terms of specificity, Youden index, PPV, NPV, DOR, accuracy, net specificity, and AUC. However, AUC performance was very similar for the top 5 SNPs (AUC = 0.879–0.881); therefore, the other SNPs would likely be informative as well. This SNP was therefore selected for entry into the final model with *APOE* e4.

Five factors that were identified as the most predictive of LOAD risk in the final (point-based) model, along with the coefficient values and numbers of points for each predictor, are shown in Table 4. In the point-based model, total risk scores ranged from 0 to 18, with a mean of 6.2 (standard deviation = 5.1). Key binary predictors included age (7 points if age >75), sex (1 point for women), years of education (6 points for ≤6 years), *APOE* e4 status (2 point for carriers), and *APOC1* rs4420638 (2 point for carriers). The total point score showed ideal prediction ability for LOAD risk [AUC = 0.87; 95% confidence interval (CI) = 0.83–0.90]. Validation of the final model using leave-one-out cross-validation (LOOCV) techniques estimated optimism as 0.004 (AUC corrected for optimism = 0.866). The mean age of the study participants was 75 years, and the predictive ability of the model was ideal with slight differences in participants aged <75 years (AUC = 0.87) and ≥75 years (0.81).

**Figure 1:**
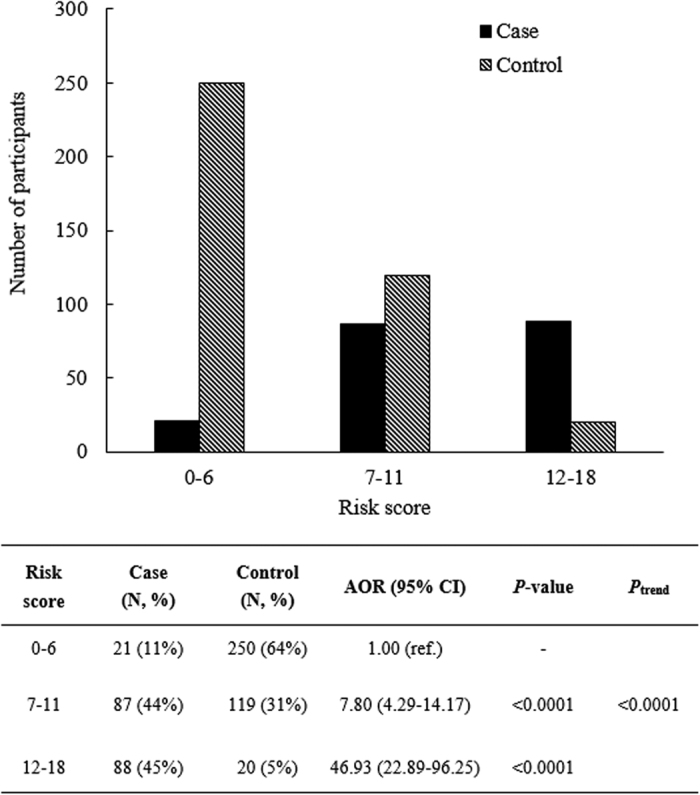
Distribution of late-onset Alzheimer’s disease cases and controls with low, moderate, and high risk.

When participants were grouped based on their risk scores, 11% of the cases showed low risk (0–6 points, n = 21), 44% of the cases showed moderate risk (7–11 points, n = 87), and 45% of cases showed high risk (12–18 points, n = 88, Fig. 1). In addition, compared with the low-risk group, the moderate- and high-risk groups showed significantly increased risks of LOAD (AOR = 7.80 and 46.93, respectively; *P*_trend_ < 0.0001, Fig. 1).

## Discussion

Five SNPs for predicting LOAD risk were identified in stage 1 (training set) by using performance metrics. These SNPs were located at four genes, including apolipoprotein C-I (*APOC1*) for rs4420638, carboxypeptidase-2 (*CPXM2*) for rs2362967, coiled-coil domain containing 81 (*CCDC81*) for rs10501617, and zinc finger protein521 (*ZNF52*1) for rs7230380 and rs12965520. If SNPs were sorted by *P-*value regardless of statistical significance, four of the top SNPs identified by *P-*value (*CPMX2* rs2362967, *APOC1* rs4420638 and *ZNF521* rs7230380 and rs12965520) were identical to the top five SNPs identified by performance metrics (Table 2). However, in both stages 1 and 2, none of the 500,941 SNPs reached statistical significance (based on *P*-value < 10^−7^) after correction for multiple tests.

*APOE* e4 is a strong risk factor of LOAD^6^; however, previous studies have indicated that its sensitivity was low (approximately 0.40)^8^,^31^, and this finding was confirmed in the current study (0.50 in stage 1 and 0.37 in stage 2). In an attempt to resolve this issue, simultaneous testing using *APOE* e4 and SNPs selected from the training set was applied in the present study. Among the top five SNPs, *APOC1* rs4420638 showed the best overall performance across different indices (criteria were defined in the Materials and Methods section). In the Caucasian population, *APOC1* rs4420638 and *APOE* rs429358 were in strong LD (D’ = 0.96, R^2^ = 0.72) based on the genotype data from the 1000 Genome Project (http://analysistools.nci.nih.gov/LDlink/?tab=home). However, these two SNPs were not in strong LD (D’ = 0.6, R^2^ = 0.012) in our population, and *APOC1* rs4420638 remained significant conditioning on *APOE* e4 status (adjusted odds ratio = 8.9, 95% CI = 3.0–26.2). The discrepancy may result from different ethnic groups. Therefore, the inclusion of *APOE* e4 status and *APOC1* rs4420638 does not indicate the same signal in this population. Point-based genetic model showed that elders belonging to moderate-risk (risk score = 7–11) and high-risk (score = 12–18) groups presented significantly increased risk of LOAD (AOR = 7.80 and 46.93, respectively; *P*_trend_ < 0.0001, Fig. 1) compared with elders with low risk scores (0–6). Although addition of *APOC1* rs4420638 did not significantly improve the prediction ability of the model, simultaneous testing with *APOE* e4 significantly increased the sensitivity of *APOE* e4 from 0.50 to 0.78, which indicates simultaneous testing could have useful clinical and public health implications.

Although the top five SNPs selected by performance metrics did not reach statistical significance based on traditional *P*-values, they have been directly or indirectly related to LOAD as discussed below. A GWAS in Caucasians found that *APOC1* rs4420638 was associated with LOAD risk^32^, which is consistent with our findings. *APOC1* is produced by astrocytes^33^ and regulates lipoprotein metabolism via its interaction with *APOE*^34^ by masking or altering the conformation of *APOE* on lipoprotein particles^35^. In addition, an animal study showed that apoC-1 may affect cognitive functions by lowering the expression of *apoE* or offsetting the effects of *apoE* on lipid distribution in the brains of mice^36^.

The SNP rs236967 is located on the *CPXM2* gene, which plays an important role in synaptic integrity and remodeling, cell adhesion^37^,^38^, and upregulation of clusterin (*CLU*), a gene previously linked to LOAD^39^. *CPXM2* is also related to AD^40^, Parkinson’s disease, and schizophrenia^38^,^41^. However, the only epidemiologic study to investigate this gene did not identify a significant association with LOAD^42^, probably because of the moderate effects of this SNP.

*CCDC81* has been associated with colorectal cancer^43^, but direct evidence to link this gene with LOAD is not available. Recent studies found that AD risk was inversely associated with cancer risk because of upregulation of oxidative phosphorylation in AD and glycolysis in cancer^44^,^45^,^46^. Therefore, the observed association between *CCDC81* rs10501617 and LOAD risk in this study (AOR = 3.3, Table 2) may be a result of this indirect relationship.

Both mice and human studies have shown that *ZNF521* plays a role in sustaining neural differentiation, regulating neural differentiation in stem cells and brain development^47^,^48^. These findings may explain the associations between *ZNF521* rs7230380 and rs12965520 and LOAD in the present study (AOR = 3.3), which have not been previously reported. Because of the complexity of AD, the mechanism underlying the modulation of disease progression by multiple genes remains to be elucidated. *BHLHB2* rs2137946 and rs2137947 were among the top SNPs selected by traditional *P-*values if no correction of multiple tests was applied. However, because they were not identified by the performance metrics and did not reach statistical significance after Bonferroni correction, their mechanisms are not described here.

The strengths of this study are as follows. First, this study compared SNPs selected by traditional *P-*values, which suffer from high false-positive and false-negative results^49^, with those selected by performance metrics. The application of performance metrics appears to allow for greater inclusion of factors with moderate effects and helps balance the risks, benefits, and costs of genetic markers for application in public health or clinical units^30^. Second, this study used extensive matching and restriction in stage 1, thereby allowing greater statistical efficiency, reducing the sample size, and subsequently lowering the cost of microarray assays. Third, simultaneous testing using *APOE* e4 and *APOC1* rs4420638, the SNP identified by performance metrics, significantly enhanced the low sensitivity of *APOE* e4 from 0.50 to 0.78 and thus made the genetic model more applicable.

This study presents a number of limitations. This work is a case-control study and may suffer from recall bias. However, a high concordance rate was observed between self-reports and medical record-confirmed vascular disease based on a random sample of 5% of all participants in the study. In addition, previous studies showed that participants’ awareness of major health issues diagnosed by physicians tended to be correct^50^,^51^,^52^. Finally, the matching ratio for cases to controls is sub-optimal (<1) because of the multiple matching criteria applied, which allowed us to control for several covariates at a time but also limited the number of controls who met the criteria.

To the best of our knowledge, this study is the first to compare SNPs for LOAD prediction selected by performance metrics with those selected by traditional *P*-values. Simultaneous testing using *APOC1* rs4420638 and *APOE* e4 to predict LOAD risk significantly improved the low sensitivity of *APOE* e4. A point-based genetic model based on these two SNPs and important covariates successfully differentiated elders with a low, moderate, and high risk of LOAD. Our findings revealed that performance metrics are an excellent alternative for identifying SNPs for disease prediction and are highly applicable for creating genetic tests with public health and clinical implications.

## Materials and Methods

### Study population

The present work was a two-stage case-control study including 294 mild to moderate LOAD cases and 503 controls recruited from the neurology clinics of three teaching hospitals in northern Taiwan from 2007 to 2010. All participants were aged 65 years or older. Each participant provided blood samples for genotyping and microarray analysis. Participants were excluded (n = 84) if blood samples were not obtained or if they had any of the following conditions or diseases: depression, Parkinson’s disease, hemorrhagic stroke, cerebral infarction, and/or organic brain tumors. After exclusion, a total of 251 LOAD cases and 462 controls were included for statistical analysis.

In stage 1 (training set), LOAD cases and controls were matched in terms of age (±5 years), gender, birthplace of parents/grandparents, and comorbidities (hypercholesterolemia, hypertension, DM, and head injury). Participants were further selected based on the following restriction criteria: 18.5 kg/m^2^ < BMI < 27 kg/m^2^ and no history of cigarette smoking. In total, 55 matched pairs (55 LOAD cases and 39 controls; one control may have matched with more than one LOAD case) were selected for genome-wide microarray scans. For comparison, five SNPs with the best performance in the performance metrics (SNPs with the largest number of indices with the highest performance metrics) and the smallest *P-*values (traditional approach) were selected. These SNPs were genotyped in another dataset (stage 2: 196 LOAD cases and 423 controls) for validation.

### Performance metrics

Performance metrics estimated in this study included clinical validity [*sensitivity* = *TP*/*(TP* + *FN) and specificity* = *TN*/*(FP* + *TN)*], Youden index (*sensitivity* + *specificity* − *1*), positive predictive value [PPV = *TP*/*(TP* + *FP)*], negative predictive value [NPV = *TN*/*(FN* + *TN)*], diagnostic odds ratio [DOR = (TP/FP)/(FN/TN)], accuracy [(*TP* + *TN*)/(TP + *TN* + *FP* + *FN*)], net sensitivity [(*sensitivity of APOE e4* + *sensitivity of selected SNP*) − (*sensitivity of APOE e4* × *sensitivity of selected SNP*)], net specificity (*specificity of APOE e4* × *specificity of selected SNP*) and AUC. Here TP denotes true positive, FN denotes false negative, FP denotes false positive, and TN denotes true negative.

### Ethics statement

The study protocol was approved by the Institutional Review Boards of National Taiwan University Hospital, En Chu Kong Hospital, and Cardinal Tien Hospital. Written informed consent was obtained from each study participant. Consent from the legal guardian/next of kin was obtained when patients had serious cognitive impairment. This study complies with the World Medical Association Declaration of Helsinki.

### Covariates

A self-reporting questionnaire (Table S1) was administered to collect information on demography, lifestyle (e.g., cigarette smoking and alcohol consumption), and disease comorbidity (e.g., hypertension, DM, cardiovascular disease, and hypercholesterolemia).

### Assessment of Alzheimer’s disease

A neurologist at each hospital diagnosed potential dementia cases. The Mini-Mental State Examination^53^ and Clinical Dementia Rating^54^ were used to evaluate cognitive function. The diagnosis of probable dementia was evaluated using the Diagnostic and Statistical Manual of Mental Disorders, Fourth Edition^55^. Head magnetic resonance imaging scans and computed tomography were taken to exclude participants with organic lesions. LOAD diagnosis was based on the National Institute of Neurological and Communicative Disorders and Stroke and the Alzheimer’s Disease and Related Disorders Association Alzheimer’s Criteria^56^. Controls with complete independence in activity of daily living and instrumental activity of daily living were assessed using a Short Portable Mental Status Questionnaire^57^, and those with possible cognitive impairment and other mental disorders were excluded from this study.

### Collection and pretreatment of biospecimens

Blood samples were collected from each participant in tubes containing sodium EDTA for genotyping. After centrifugation, genomic DNA was extracted from the buffy coat using a QuickGene-Mini 80 system (Fujifilm, Tokyo, Japan) and then stored at −80 °C. To ensure the quality of the microarray genotyping assay, all DNA samples were required to meet the following criteria: OD_260_/OD_280_ (DNA quality) = 1.8–2.0, OD_260_/OD_230_ (remaining organic solvent) >1.5 and zero fragments of genomic DNA.

### Genotyping assays

For stage 1, the Axiom™ Genome-Wide CHB 1 Array Plate (Affymetrix Inc., Santa Clara, California), which includes 563,746 SNPs, was used for genotyping. This array chip is optimized for the best coverage of common variants in the Chinese population. For the microarray data, SNPs were excluded based on the following quality control criteria: (1) genotyping success rate <90%, (2) call rate <98%, (3) MAF <0.05, or (4) *P-*value of HWE tests in controls <0.0001. In total, 511,718 SNPs were included after frequency and genotyping pruning. Because frequencies differed between SNPs on sex and autosomal chromosomes, SNPs located on sex chromosomes were also excluded from analysis. After further exclusion of 10,877 SNPs, 500,941 SNPs remained for further analysis.

*APOE* e4 genotypes were determined by rs429358 and rs7412^58^. Because these two SNPs were not included in the array chip, they were genotyped together with the top five SNPs selected by performance metrics at stage 1 for all participants (stages 1 and 2) by employing TaqMan Genomic Assays using an ABI 7900HT Fast Real-time PCR system (Applied Biosystems Inc., CA, USA). We selected the top 5 SNPs because these SNPs showed moderate association with the outcome and the inclusion of more SNPs may not improve AD prediction ability. In addition, based on the principle of parsimony for model building and better generalizability, we avoided including too many predictors. The genotyping call rate was greater than 95% for SNPs determined by the TaqMan assays. The internal quality control obtained from 5% of the samples in duplicate had a concordance rate of 100%.

### Statistical analyses

To compare the distribution between cases and controls, Student’s *t*-tests and Mann-Whitney U tests were used for normally and non-normally distributed continuous variables, respectively. Normality of continuous variables were checked by visual inspection if there was strong deviation from the diagonal line on QQ plots. Chi-square tests were used for categorical variables.

The PLINK program (http://pngu.mgh.harvard.edu/~purcell/plink/) was used to estimate genotype frequency, *P-*value of the HWE test, and MAF for each SNP. Instead of using an additive model, a dominant genetic model was used to build a contingency table. Performance metrics, e.g., sensitivity, specificity, PPV and NPV and others, were estimated by classifying the genotype data into positive (carriers of a variant allele) and negative (wildtype) disease state predictions. Five SNPs with the best performance in the performance metrics were selected for genotyping in stage 2 (validation set). Traditional *P*-values and AORs were calculated for comparison. Simultaneous testing of *APOE* e4 and each of the five SNPs selected in stage 1 was performed to estimate the net sensitivity and net specificity. The SNP with the best overall performance in the performance metrics was used to construct the genetic model for predicting LOAD. *APOE* e4 was forced into the genetic model because it is a well-known genetic risk factor for LOAD.

Multivariable logistic regression models were used to estimate AORs and 95% CIs for LOAD cases in the dominant genetic model. For stage 1 (training set), the regression model was conditioned on the matched set. For stage 2 (validation set), the study participants were conditioned on an age interval of 5 years to control for the confounding effect of age, i.e., cases and controls were compared within each stratum in the multivariable analysis. Continuous age variable was also adjusted in the model to control for the residual confounding within each 5-year age stratum. In the final genetic model, age (65–75 and >75 years old), sex, years of education (>6 and ≤6 years), *APOE* e4 status (carriers vs. non-carriers), and SNPs identified by performance metrics were adjusted. Each variable was then assigned a point value by dividing its model coefficient value with the coefficient for sex, i.e., the smallest coefficient for a dichotomous variable in the final model, rounding up to the nearest integer. This point-based approach has previously been successfully used to develop clinical prediction tools^59^,^60^. In addition, the AUC statistic was also used for model discrimination, where AUC ≥ 0.7 indicates acceptable discriminative ability. LOOCV was used to evaluate the internal validity of the final model.

For comparison with traditional GWAS results, a Manhattan plot and a QQ plot were produced using PLINK and R (http://www.r-project.org/), respectively, to assess whether the *P-*values deviated from the expected distribution (i.e., H_0_: no association between SNPs and LOAD). SAS version 9.4 (SAS Institute, Cary, NC) was used to conduct all statistical analyses, and all statistical tests were two-sided.

## Additional Information

**How to cite this article**: Chen, Y.-C. *et al*. Performance Metrics for Selecting Single Nucleotide Polymorphisms in Late-onset Alzheimer’s Disease. *Sci. Rep.* **6**, 36155; doi: 10.1038/srep36155 (2016).

**Publisher’s note:** Springer Nature remains neutral with regard to jurisdictional claims in published maps and institutional affiliations.

## Supplementary Material

Supplementary Information

## Figures and Tables

**Table 1:** Demographics of the study population.

Variables	Stage 1: Training set (N = 94)			Stage 2: Validation set (N = 619)		
	LOAD	Control	*P-*value	LOAD	Control	*P-*value
	N = 55	N = 39		N = 196	N = 423	
	**Mean ± SD**			**Mean ± SD**		
Age (years)	78.3 ± 5.6	75.4 ± 4.8	**0.01**	79.9 ± 6.5	73.1 ± 5.9	**<0.0001**
Years of education	8.3 ± 5.5	11.4 ± 4.4	**0.004**	7.9 ± 5.0	12.7 ± 4.1	**<0.0001**
BMI (kg/m^2^)	23.3 ± 1.7	23.4 ± 1.9	0.75	22.8 ± 3.6	24.2 ± 3.1	**<0.0001**
	**N (%)**			**N (%)**		
Female	38 (69)	24 (62)	0.45	121 (62)	210 (50)	**0.01**
*APOE* e4 carriers	27 (50)	4 (11)	**<0.0001**	72 (37)	59 (15)	**<0.0001**
Alcohol drinking	4 (7)	1 (3)	0.32	27 (14)	50 (12)	**0.50**
Ever smoking	—	—	—	56 (29)	75 (18)	**<0.002**
Cardiovascular disease	11 (20)	7 (18)	0.85	50 (26)	137 (33)	0.08
Stroke	2 (4)	1 (3)	0.77	7 (4)	5 (1)	0.05
Hypertension	24 (44)	18 (46)	0.81	74 (38)	236 (56)	**<0.0001**
DM	—	—	—	48 (24)	67 (16)	**0.01**
Hypercholesterolemia	—	—	—	46 (24)	141 (33)	**0.02**

Abbreviations: **LOAD,** late-onset Alzheimer’s disease; **SD,** sample standard deviation; **BMI,** body mass index; ***APOE***, apolipoprotein E; **DM,** diabetes mellitus.

The *P-*value was obtained by comparing the distribution of LOAD with the controls.

Numbers in bold indicate significant differences between cases and controls.

Because of restriction and matching in the training set (stage 1), some variables showed empty cells in the table.

**Table 2:** Comparison between two *APOE* SNPs and the top SNPs selected by performance metrics or traditional measures in stage 1 and stage 2.

Gene	SNP	Performance metrics										Traditional measures	
		Sensitivity	Specificity	Youden	PPV	NPV	DOR	Accuracy	Net sensitivity^a^	Net specificity^a^	AUC (95% CI)	*P-*value^b^	AOR (95% CI)
Stage 1 (Training set, N = 94)													
* APOE* e4	rs429358 & rs7412^*c*^	0.50	0.89	0.39	0.87	0.56	8.5	0.66	—	—	0.835 (0.749–0.922)	3.0 × 10^−4^	11.68 (3.13–43.53)
* CCDC81*	rs10501617	*0.71*	*0.73*	*0.43*	*0.78*	*0.64*	*6.4*	*0.72*	*0.85*	*0.65*	***0.888 (0.822–0.955*)**	5.8 × 10^−4^	3.3 (1.6–6.5)
* CPXM2*	rs2362967	***0.89***	*0.49*	*0.38*	*0.71*	*0.76*	*7.8*	*0.72*	***0.95***	*0.44*	*0.873 (0.801–0.944*)	*3.4 × 10^−5^*	*3.6 (1.9–6.6*)
* APOC1*	rs4420638	*0.56*	***0.90***	***0.46***	***0.89***	*0.60*	***11.6***	*0.71*	*0.78*	***0.81***	**0.873 (0.799–0.947*)*	***6.2 × 10***^***−6***^	***8.9 (3.0–26.2*)**
* ZNF521*	rs7230380^d^	***0.89***	*0.53*	*0.42*	*0.72*	***0.78***	*9.0*	***0.74***	***0.95***	*0.47*	*0.879 (0.812–0.947*)	*1.3 × 10^−4^*	*3.3 (1.8–6.1*)
* ZNF521*	rs12965520^d^	***0.89***	*0.53*	*0.42*	*0.72*	***0.78***	*9.0*	***0.74***	***0.95***	*0.47*	(*0.812–0.947*)	*1.3 × 10^−4^*	*3.3 (1.8–6.1*)
* BHLHB2*	rs2137946	0.38	0.18	−0.44	0.39	0.17	0.13	0.29	0.43	0.15	0.860 (0.781–0.938)	*4.8 × 10^−5^*	*0.4 (0.2–0.7*)
* BHLHB2*	rs2137947	0.38	0.18	−0.44	0.39	0.17	0.13	0.29	0.43	0.15	0.862 (0.785–0.939)	*4.8 × 10^−5^*	*0.4 (0.2–0.7*)
Stage 2 (Validation set, N = 619)													
* APOE* e4	rs429358 & rs7412^*c*^	0.37	0.85	0.22	0.55	0.73	3.26	0.69	—	—	0.879 (0.850–0.909)	<0.0001	3.99 (2.36–6.75)
* CCDC81*	rs10501617	0.44	0.54	−0.02	0.31	0.67	0.91	0.51	0.64	0.46	0.879 (0.849–0.909)	6.3 × 10^−1^	0.92 (0.64–1.31)
* CPXM2*	rs2362967	**0.73**	0.30	0.03	0.33	0.71	1.16	0.44	**0.83**	0.25	0.879 (0.850–0.909)	6.4 × 10^−1^	0.93 (0.68–1.28)
* APOC1*	rs4420638	0.42	**0.79**	**0.21**	**0.48**	**0.75**	**2.73**	**0.67**	0.63	**0.67**	**0.881 (0.852–0.911)**	**9.0 × 10**^**−2**^	1.85 (0.90–3.79)
* ZNF521*	rs7230380	0.71	0.25	−0.04	0.30	0.65	0.82	0.40	0.82	0.21	0.879 (0.850–0.909)	2.4 × 10^−1^	**3.07 (0.47–20.1)**
* ZNF521*	rs12965520	0.71	0.25	−0.03	0.31	0.66	0.85	0.40	0.82	0.22	0.879 (0.850–0.909)	2.8 × 10^−1^	0.36 (0.06–2.32)

Abbreviations: **SNP,** single nucleotide polymorphism; **PPV,** positive predictive value; **NPV,** negative predictive value; **DOR,** diagnostic odds ratio; **AOR,** adjusted odds ratio; **AUC,** area under the receiver operating characteristic curve; **CI,** confidence interval.

^a^Net sensitivity and net specificity were obtained for each selected SNP and *APOE* e4 for simultaneous screening purposes.

^b^None of the SNPs reached statistical significance (*P* < 10^−7^) after correction for multiple tests using the traditionally measured *P-*value.

^c^*APOE* e4 status was determined by rs429358 & rs7412; the performance metrics estimation was based on *APOE* e4 carriers versus non-carriers.

^d^Because of ties (*P* = 1.3 × 10^−4^ for *ZNF521* rs7230380 and rs12965520), 6 SNPs were selected using the traditional *P*-value.

Except for *APOE* e4, the performance metrics or traditional measures for the remaining SNPs were estimated based on dominant genetic models.

For SNPs selected based on performance metrics or *P*-value, numbers in bold indicate the highest value of the corresponding indices or the lowest for *P*-value.

Italicised numbers indicate the top 5 SNPs selected by performance metrics and the top 6 SNPs (because of ties) selected by traditional *P*-values (regardless of statistical significance after correction for multiple tests), respectively.

*BHLHB2* rs2137946 and rs2137947 were selected using traditional *P*-value regardless of statistical significance. Because these two SNPs were not identified by performance metrics, they were not genotyped in stage 2.

Numbers in bold indicate the largest value of each performance matrix or traditional measure.

**Table 3:** Characteristics of 2 *APOE* SNPs and the top 5 SNPs selected by performance metrics in stage 2 (N = 619).

Gene	SNP	Nucleotide change	Chromosome/Location	HapMap or dbSNP MAF			Controls (n = 423)		Cases (n = 196)	
				CEU	CHB	JPT	MAF	HWE*P-*value	MAF	HWE *P-*value
*APOE*	rs429358 (*APOE*112)	T → C	19/exon	0.15*	0.00	0.01	0.08	0.68	0.23	0.27
*APOE*	rs7412 (*APOE*158)	C → T	19/exon	0.08*	0.11	0.05	0.08	0.22	0.06	0.97
*APOC1*	rs4420638	G → A	19/3′UTR	0.18	0.12	0.07	0.11	0.93	0.23	0.75
*CCDC81*	rs10501617	T → C	11/intronic	0.40	0.20	0.16	0.27	0.37	0.26	0.64
*ZNF521*	rs7230380	A → C	18/intronic	0.47	0.49	0.50	0.50	0.90	0.48	0.36
*ZNF521*	rs12965520	T → C	18/intronic	0.50	0.45	0.50	0.50	0.58	0.48	0.33
*CPXM2*	rs2362967	C → T	10/intronic	0.26	0.50	0.58	0.48	0.03	0.48	0.78

Abbreviations: **SNP,** single nucleotide polymorphism; **UTR,** untranslated region; **CEU,** Utah residents with Northern and Western European ancestry from the CEPH collection; **JPT,** Japanese in Tokyo, Japan; **CHB,** Han Chinese in Beijing; **MAF,** minor allele frequency; **HWE,** Hardy-Weinberg equilibrium test.

*MAF data were obtained from dbSNP because the data were not available from the HapMap Project.

**Table 4:** Final genetic model for predicting late-onset Alzheimer’s disease.

Variables	Coefficient	Point
Age groups		
* *65–75	0	0
* *>75	2.42	7
Sex		
Men	0	0
Women	0.37	1
Education		
>6 years	0	0
≤6 years	2.11	6
*APOE* e4 status		
Non-carriers	0	0
Carriers	0.91	2
*rs4420638 (APOC1*)		
Non-carriers	0	0
Carriers	0.64	2

Total range		0–18
Mean score ± SD		6.2 ± 5.1
AUC (95% CI)		0.87 (0.83–0.90)
Corrected for optimism		0.866

Abbreviations: **SD,** sample standard deviation**; AUC,** area under the receiver operating characteristic curve; **CI,** confidence interval.

The correction for optimism was performed by leave-one-out cross-validation (LOOCV) in stage 2 (validation set).
