# Supplementary material for: Performance Metrics for Selecting Single Nucleotide Polymorphisms in Late-onset Alzheimer’s Disease
Source: Sci Rep. 2016 Nov 2;6:36155. doi: 10.1038/srep36155 (PMC5090242; doi:10.1038/srep36155)
Supplement: Supplementary Information [file srep36155-s1.pdf]

Performance Metrics for Selecting Genetic Polymorphisms in Late-onset Alzheimer's Disease

Short title: Performance metrics of Genetic Markers for Alzheimer's Disease

Yen-Ching Chen[1,2,3*], Chi-Jung Hsiao[1], Chien-Cheng Jung[1], Hui-Han Hu[4], Jen-Hau Chen[1,5], Wen-Chung Lee[1,2,3], Jeng-Min Chiou[6], Ta-Fu Chen[7], Yu Sun[8], Li-Li Wen[9], Ping-Keung Yip[10,11], Yi-Min Chu[12], Chien-Jen Chen[4*], Hwai-I Yang[4,13*]

1 Institute of Epidemiology and Preventive Medicine, College of Public Health, National Taiwan University, Taipei, Taiwan;

2 Department of Public Health, College of Public Health, National Taiwan University, Taipei, Taiwan;

3 Research Center for Genes, Environment and Human Health, College of Public Health, National Taiwan University, Taipei, Taiwan;

4 Genomics Research Center, Academia Sinica, Taipei, Taiwan;

5 Department of Geriatrics and Gerontology, National Taiwan University, Taipei, Taiwan;

6 Institute of Statistical Science, Academia Sinica, Taipei, Taiwan;

7 Department of Neurology, National Taiwan University Hospital, Taipei, Taiwan;

8 Department of Neurology, En Chu Kong Hospital, Taipei, Taiwan;

9 Department of Laboratory Medicine, En Chu Kong Hospital, Taipei, Taiwan;

10 Center of Neurological Medicine, Cardinal Tien Hospital, Taipei, Taiwan;

11 School of Medicine, Fu-Jen Catholic University, New Taipei City, Taiwan;

12 Department of Laboratory Medicine, Cardinal Tien Hospital, Taipei, Taiwan;

13 Institute of Clinical Medicine, National Yang-Ming University, Taipei, Taiwan.

* Address for reprint requests and correspondence: Dr. Yen-Ching Chen, No. 17 Xu-Zhou Road, Institute of Epidemiology and Preventive Medicine, College of Public Health, National Taiwan University, Taipei, Taiwan. Telephone: 886-2-3366-8019. Fax: 886-2-2351-1955. E-mail: karenchen@ntu.edu.tw

Dr. Chien-Jen Chen, Genomics Research Center, Academia Sinica, Taipei, Taiwan. E-mail:

chencj@gate.sinica.edu.tw

Dr. Hwai-I Yang, Genomics Research Center, Academia Sinica, Taipei, Taiwan. Telephone: 886-2-2787-1308. E-mail: hiyang@gate.sinica.edu.tw

Supplementary Figure and Table Legends

Figure S1. Quantile-quantile plots of the observed *P*-values for the association of each SNP and LOAD risk

Figure S2. Manhattan plot for 500,941 single nucleotide polymorphisms of the study population

Table S1. Questionnaire for collecting demographic information and disease comorbidities

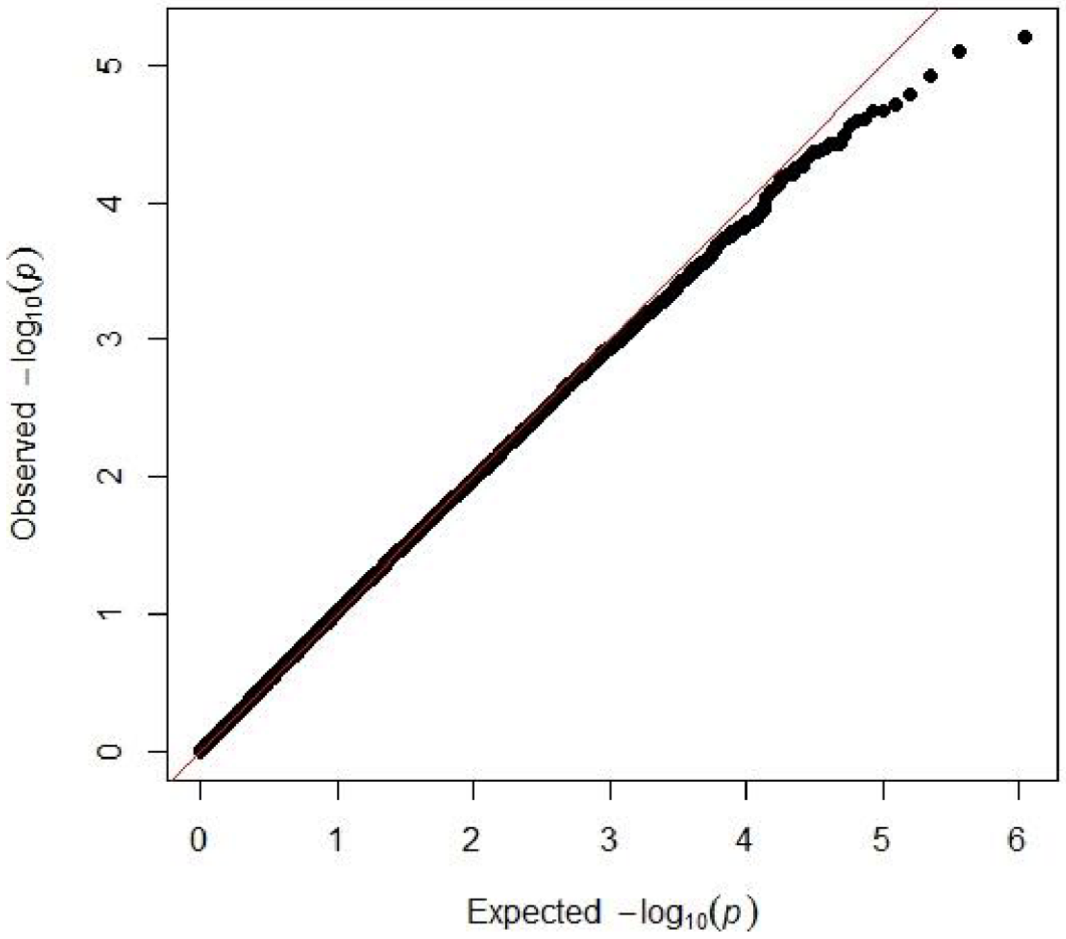

Figure S1. Quantile-quantile plots of the observed *P*-values for the association of each SNP and LOAD risk

The QQ plot reflects several SNPs with an observed chi-square *P*-value smaller than the expected *P*-value at 10^{-5}, but none of them reach statistical significance ($P < 10^{-7}$). The solid diagonal line indicates the expected *P*-value (i.e., null hypothesis of no association between SNPs and LOAD) with a slope of 1 and an intercept of 0.

Abbreviations: QQ, Quantile-Quantile; **SNP,** single nucleotide polymorphism.

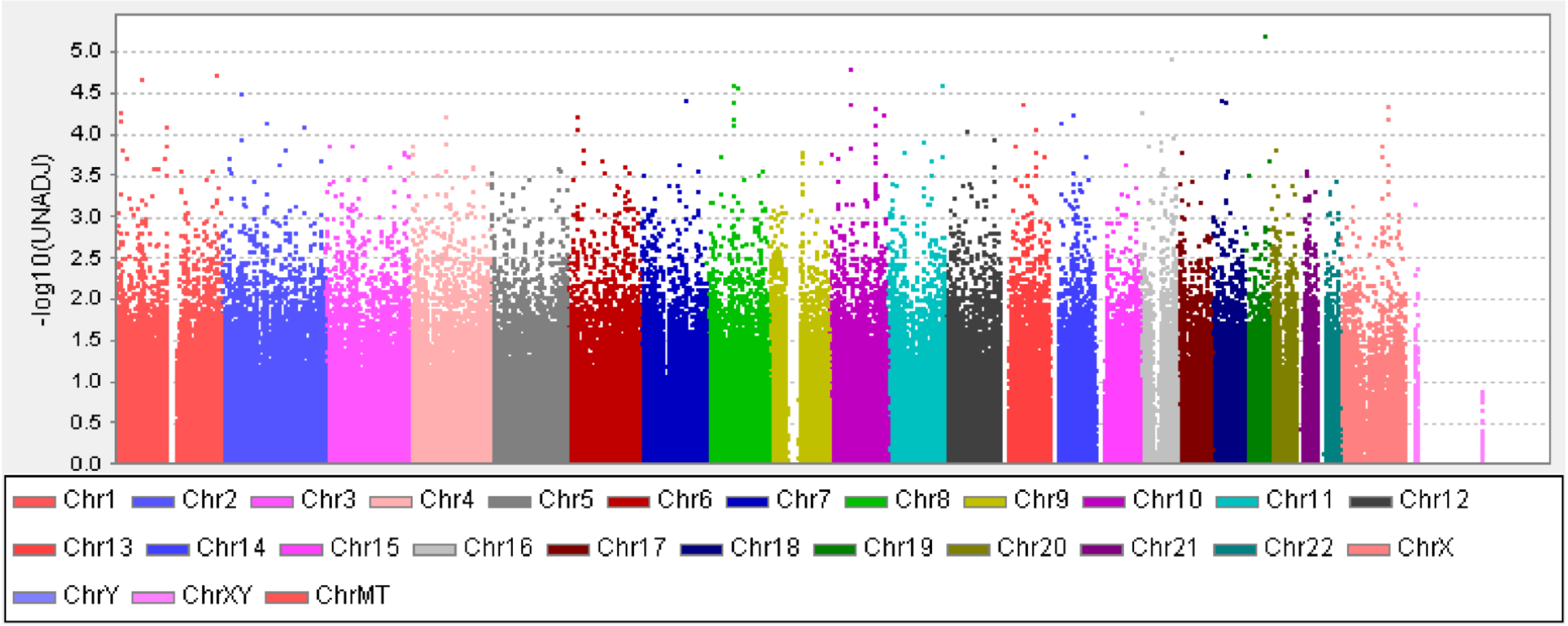

Figure S2. Manhattan plot for 500,941 single nucleotide polymorphisms of the study population

Abbreviations: CHR, chromosome; **SNP,** single nucleotide polymorphism.

The Y-axis indicates the $-\log_{10}P$ of the Cochran-Armitage trend test.

The X-axis indicates the corresponding position of each SNP on the chromosome.

Table S1. Questionnaire for collecting demographic information and disease comorbidities

**Basic information:**		
( )	1	Gender: 0 Female 1 Male
( )	2	Years of education (since elemental school)
( )	3.1	What is the birthplace of your father? ________
( )	3.2	What is the birthplace of your mother? ________
( )	3.3	What is the birthplace of your grandparents? ________
( )	3.4	What is the birthplace of your maternal grandparents? ________
( )	4	Work/occupation status: 1 Unemployment 2 Housekeeper 3 Labor 4 Skilled worker 5 Captain or self-employed worker 6 Clerical worker or salesman 7 Technician or professional 8 Supervisor or entrepreneur 999 Unknown
( )	5	Annual household income (New Taiwan Dollar): 1 < 300,000 2 300,000-800,000 3 800,000-1,000,000 4 > 1,000,000 999 Unknown
( )	6	Current weight（Kilogram）
( )	7	Current height（Centimeter）
( )	8	Menopause: 0 Not yet 1 Yes 2 Not applicable 999 Unknown
( )	9	Religious affiliation: 0 No 1 Christian 2 Catholicism 3 Taoism 4 Buddhism 5 Islam 6 Other
( )	10	Family support: 1 Live with spouse and kid(s) 2 Live with spouse 3 Live with kid(s) 4 Live with grandchildren or other relatives 5 Live alone 6 Live at nursing home
( )	11	Marital status: 1 Un-married 2 Widowed 3 Divorced/separated 4 Married 5 Co-habit
( )	12	Alcohol drinking: 0 No 1 Yes
( )	13	Cigarette smoking: 0 No on average 1 < 1 pack per day 2 1-2 packs per day 3 > 2 packs per day
( )	14	Hypertension: 0 No 1 Clinic visit and regular medication 2 Clinic visit but without regular medication
( )	15	Diabetes mellitus: 0 No 1 Clinic visit and regular medication 2 Clinic visit but without regular medication
( )	16	Stroke: 0 No 1 Clinic visit and regular medication 2 Clinic visit but without regular medication
( )	17	Hyperlipidemia: 0 No 1 Clinic visit and regular medication 2 Clinic visit but without regular medication
( )	18	Heart diseases: 0 No 1 Clinic visit and regular medication 2 Clinic visit but without regular medication
